# Enhancing Dementia Awareness and Screening, and Reducing Stigmatizing Attitudes towards Dementia in Urban China: The Role of Opinion Leader Intervention in Community-Dwelling Older Adults

**DOI:** 10.31083/AP38857

**Published:** 2025-03-07

**Authors:** Jing Wang, Hai-Yan Zeng, Jing-Xing Gao, Bao-Liang Zhong

**Affiliations:** ^1^Department of Psychiatry, Wuhan Mental Health Center, 430012 Wuhan, Hubei, China; ^2^Department of Public Health, Wuhan Hanyang District Jiangdi Street Community Health Service Center, 430050 Wuhan, Hubei, China

**Keywords:** dementia awareness, stigmatizing attitudes, screening, opinion leader, older adults

## Abstract

**Background:**

Dementia in China is increasingly burdensome yet remains underrecognized and undertreated due to low awareness and persistent stigma. Community-based strategies are urgently needed to address these barriers. By using real-world data from an 18-month dementia campaign in Wuhan, we retrospectively evaluated the feasibility and efficacy of opinion leader intervention (OLI), a novel, community-driven approach, in improving dementia knowledge, reducing stigma, and promoting screening among older urban adults.

**Methods:**

Starting in August 2023, a three-phase campaign was launched, targeting 3550 residents aged ≥60 years in the Jiangdijie community, Wuhan. The pre-intervention phase (6 months) included monthly expert-led dementia education lectures at a senior center (30–60 attendees/session). The traditional intervention phase (next 6 months) involved distributing brochures in public areas and doubling lecture frequency. The final OLI phase (6 months) engaged 19 trained opinion leaders to disseminate knowledge, encourage screening, and model preventive behaviors during daily interactions. Free dementia screening was available throughout the campaign. Outcomes—dementia knowledge scores, stigma-related attitude scores, and screening participation rates—were assessed via samples randomly drawn from the elderly residents at the end of each phase (T0: n = 100, T1: n = 117, T2: n = 100).

**Results:**

Dementia knowledge scores among older adults increased from 12.27 (T0) to 13.51 (T1), with a significant jump to 17.63 post-OLI (T2 vs T1, *p* < 0.001). Stigmatizing attitudes scores improved from 2.11 (T0) to 2.43 (T1), rising further to 2.98 at T2 (T2 vs T1, *p* = 0.010). Participation rates in dementia screening rose from 18.0% (T0) to 23.9% (T1), surging to 46.0% post-OLI (T2 vs T1, *p* < 0.001).

**Conclusions:**

The OLI was associated with marked improvements in dementia knowledge, reduced stigma, and higher screening uptake compared with traditional health education methods. These findings highlight OLI's potential as a feasible strategy to enhance dementia awareness and care in Chinese urban communities.

## Main Points

1. Compared with traditional intervention, dementia knowledge and stigmatizing attitudes towards dementia scores improved significantly following opinion leader intervention.

2. Rates of participation in dementia screening showed a notable increase, climbing from 23.9% at the end of traditional intervention to 46.0% at the end of opinion leader intervention.

3. Awareness of the dementia campaign increased from 44.0% at baseline to 47.0% after traditional intervention, and then rose substantially to 73.0% after opinion leader intervention.

4. The findings highlight the potential of opinion leader intervention as a valuable strategy for addressing dementia-related challenges in community settings.

## 1. Introduction

Because of the rapid aging in recent decades in China, dementia has become a major public health challenge, posing an enormous burden on healthcare and public social welfare systems [1, 2, 3]. Dementia management in China is characterized by very low rates of recognition and treatment as well as a high rate of diagnosis delay. For example, in a nationwide population-based survey, only 28.6% of seniors with dementia received a diagnosis, with this figure dropping to as low as 7% among rural Chinese elderly individuals [4]. Chinese patients with dementia typically experience an average delay of 2 years from symptom onset to diagnosis [5]. Most elderly Chinese individuals with dementia are diagnosed at the moderate and severe stages, missing the optimal window for early intervention. Low dementia awareness and widespread stigmatizing attitudes towards dementia among the general population are two key barriers contributing to poor recognition and prolonged delays in diagnosis [6, 7, 8]. For example, in Shanghai, a metropolitan city in China, 45.2% of residents believe that dementia is an inevitable part of aging, and 44.8% would not want others to know if a family member were diagnosed with dementia [9].

Public health programs that focus on improving dementia knowledge and reducing stigma are essential components of a comprehensive strategy to address the dementia burden [10]. This is particularly critical in settings like China, where low awareness and high stigma are significant barriers to effective dementia care. By addressing the root causes of delayed diagnosis and social exclusion, these programs not only improve individual outcomes but also contribute to a more informed, compassionate, and dementia-friendly society [11]. In public health practice, health education lectures on dementia prevention and control, promotional brochures, and science popularization videos are typically recommended to raise awareness and reduce stigma among community-dwelling older adults [12, 13]. However, due to China’s large elderly population, traditional health education approaches often require extensive resources to organize large-scale media campaigns, which can be both infeasible and ineffective due to high costs. Many older adults may lack access to or interest in lectures, brochures, or online videos. Barriers such as mobility issues and hearing and vision impairments, particularly in rural areas, may further limit the reach of these measures. In addition, traditional educational methods often fail to engage older adults in a meaningful way [14]. Passive learning through lectures or videos might not lead to increased knowledge or behavioral change [15].

Opinion leader intervention (OLI) is a public health strategy that uses the influence of trusted community members to promote health behaviors and spread information. Opinion leaders, as respected individuals, can shape attitudes, beliefs, and behaviors, inspiring others to adopt new practices [16]. According to the Diffusion of Innovations Theory, when opinion leaders embrace a new behavior, it can spread throughout their social network, leading to broader acceptance within the community [10]. Successful examples include the “TB Móvil” program in Lima, Peru, where community health workers and others promoted tuberculosis screening, significantly increasing awareness and participation [17]. In China, trained opinion market leaders discussed sexually transmitted disease/acquired immune deficiency syndrome-related issues, leading to a decline in stigmatizing attitudes [18]. These interventions demonstrate the effectiveness of leveraging opinion leaders in public health efforts.

To our knowledge, no prior studies have evaluated the efficacy of OLI in improving dementia-related knowledge, attitudes, and screening participation among community-dwelling older adults. Building on existing evidence, we hypothesized that OLI could enhance dementia awareness, reduce stigma, and increase screening uptake in this population. By using real-world data from a pilot community that adopted both traditional health education and OLI, this study retrospectively assessed the feasibility and efficacy of OLI in advancing dementia prevention and management. The ultimate goal of this study is to inform China’s *National Action Plan for Addressing Dementia in the Elderly (2024–2030)* [12], supporting nationwide efforts to achieve its dementia care objectives.

## 2. Material and Methods

### 2.1 Settings and Participants

The dementia campaign was conducted in the Jiangdijie community, an urban community in Wuhan’s Hanyang district, China. In 2023, the community comprised 6359 households and 3550 residents aged 60 years or older. While the intervention theoretically targeted all eligible older adults, logistical constraints limited full coverage. Inclusion criteria were: (1) aged ≥60 years, (2) residency in the Jiangdijie community for ≥6 months prior to the intervention, and (3) voluntary participation. Exclusion criteria included non-Chinese-speaking residents, those who moved out of the community, those who died during the intervention period, and those with severe disabilities (e.g., late-stage dementia, deafness, or speech impairments) that prevented study participation.

### 2.2 Interventions

From August 2023 to January 2025, we conducted a public health campaign promoting dementia awareness and early recognition within the Jiangdijie community. This campaign adhered to *China’s Action to Promote Prevention and Treatment of Dementia in the Elderly (2023–2025)* [13] and involved three 6-month phases: pre-intervention, traditional intervention, and OLI. During the pre-intervention phase, interventions included publicizing the campaign, free dementia consultations and screenings at the Jiangdijie community health center, and monthly specialist-led lectures on dementia (30–60 attendees per lecture). These activities, aimed at “warming up” the campaign, were not standardized. The traditional intervention phase built upon the initial efforts with increased lecture frequency to biweekly sessions, refining lecture content with practical examples, suggestions for a healthy lifestyle, and addressing common myths and misperceptions. Dementia-related promotional materials were also distributed at popular community venues.

The OLI phase identified and trained opinion leaders from various community sectors through ethnographic observations and interviews, targeting their social networks to ensure the inclusion of isolated elderly individuals. In this study, criteria for qualified community opinion leaders included influence and respect, communication skills, social connectivity, interest in public health services, willingness to learn, and diverse backgrounds [19, 20, 21]. Training for these leaders covered dementia clinical presentations, risk factors, prevention strategies, and communication skills, incorporating interactive teaching methods. A total of 25 opinion leaders were identified; ultimately, 19 were included as formal opinion leaders. This was because two failed to pass the eligibility test, two could not provide continuous community services over the following 6 months, and two did not complete the training. The 19 trained opinion leaders subsequently engaged community elders in personalized conversations about maintaining cognitive health and promoting regular dementia screenings. Opinion leaders encouraged the elderly residents to further share the health information with their neighbors, acquaintances, and friends. They also facilitated connections with local primary care physicians and maintained a feedback loop with the campaign team to refine strategies based on real-world experiences. Throughout the entire study period, free dementia screening was available at the community health center, ensuring continuous access to screening and consultation services for older adults in the community.

### 2.3 Efficacy Assessment

To evaluate the interventions’ efficacy, three representative samples (n = 120) were randomly drawn without replacement at three time points: the end of the pre-intervention phase (T0), the traditional intervention phase (T1), and the OLI phase (T2). Participants were interviewed via their preferred method, including in-person, phone, or online video chat. The study assessed three outcomes: dementia-related knowledge, stigmatizing attitudes, and participation in the dementia screening program.

Knowledge and attitudes were measured using study-specific questionnaires: a 22-item knowledge scale and a 4-item attitude scale. Both tools were developed following standardized scale development procedures. Briefly, an initial item pool (knowledge: 84 items; attitude: 10 items) was formed based on a literature review, Delphi consultations with dementia experts, and interviews with older adults. After three additional rounds of Delphi consultation, a preliminary version of the two scales (knowledge: 33 items; attitude: seven items) was generated and tested in a sample of 55 older adults. Item analysis and exploratory factor analysis (EFA) with varimax rotation were used to refine the items. This resulted in a final 22-item knowledge scale (divided into three subscales: clinical presentations, prognosis, and modifiable risk factors/prevention) and a 4-item unidimensional attitude scale. Content validity was assessed via expert ratings (eight experts; 4-point relevance scale), achieving scale-level content validity indices of 0.983 (knowledge) and 0.969 (attitude). Psychometric evaluation in 185 older adults demonstrated high internal consistency (Cronbach’s α: knowledge scale = 0.948, subscales = 0.833–0.897; attitude scale = 0.796). Structural validity was supported by EFA-derived subscales. Empirical validity was further evidenced by significantly higher scores in participants with family members diagnosed with dementia compared with those without (knowledge: 17.55 ± 5.41 vs 13.66 ± 7.23; attitude: 3.16 ± 0.99 vs 2.34 ± 1.53; both *p* < 0.001). Total scores ranged from zero to 22 (knowledge) and zero to four (attitude), with higher totals indicating greater knowledge and lower levels of stigma.

Participation in screening was evaluated by asking if participants had participated in dementia screening at the community health center in the past 6 months. Awareness of the campaign was measured by asking participants if they knew about a dementia campaign in the community. Demographic variables assessed included sex, age, education, and marital status.

**Fig. 1.**
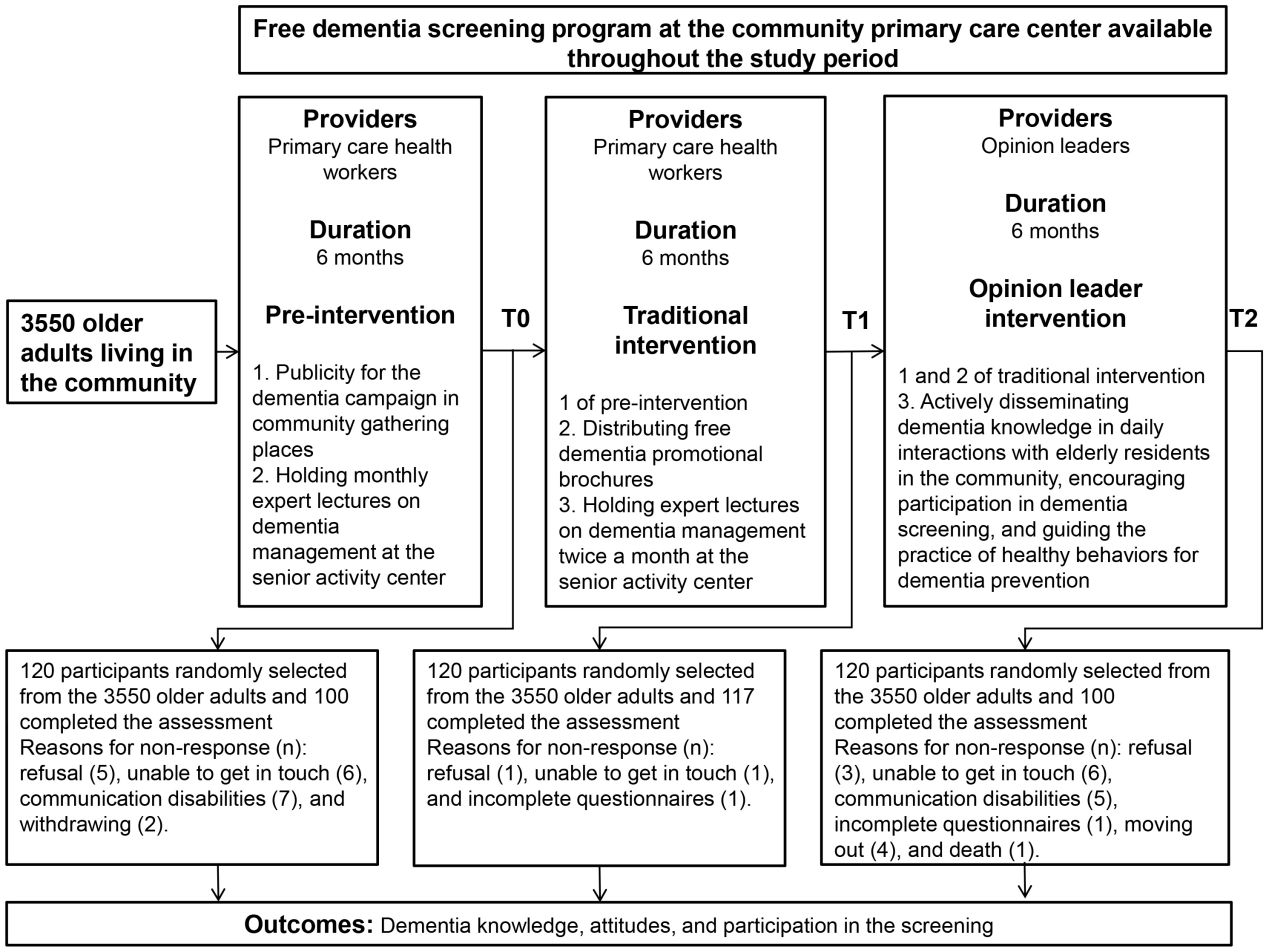
**Flowchart outlining the intervention and efficacy outcome assessment procedures of the dementia campaign**.

An overview of the interventions and efficacy outcome assessment of this study are displayed in Fig. 1.

### 2.4 Statistical Analysis

In this study, the primary outcome was the screening participation rate, with the key comparison focusing on the difference between T2 and T1 rates. Collaborating with local primary health workers, we estimated T1 and T2 screening participation rates to be 20% and 40%, respectively. Using PASS 15.0 software (NCSS, LLC., Kaysville, UT, USA), a sample size of 79 participants per group was calculated to detect this 20% proportional difference (40% vs 20%) with 80% power and a significance level of 0.05 [22]. However, to account for challenges in recruiting older adults and potential difficulties in questionnaire completion, we applied a conservative 65% anticipated response rate. Consequently, the minimum required sample size per group was adjusted to approximately 120 participants for outcome assessment.

A Chi-squared test or Fisher-Freeman-Halton exact test was used, as appropriate, to compare demographic characteristics across the three samples and assess their comparability. An independent-samples Mann-Whitney U test was used to compare the dementia knowledge and stigmatizing attitude scores between samples, while a Chi-squared test was used to compare the rates of dementia screening participation and dementia campaign awareness between samples. Statistical significance was set at *p* < 0.05 (two-sided). All analyses were conducted using SPSS software version 15.0 (SPSS Inc., Chicago, IL, USA).

## 3. Results

At T0, T1, and T2, a total of 100, 117, and 100 older adults were approached and completed the efficacy outcome assessment, respectively. Fig. 1 displays the reasons for non-response in the outcome assessment. As shown in Table 1, the three samples are comparable in terms of sex, age group, education, and marital status (*p* = 0.497–0.878).

**Table 1.** **Demographic characteristics of the three samples of older adults at the end of pre-intervention (T0), traditional intervention (T1), and opinion leader intervention (T2), n (%)**.

Characteristic		T0 (n = 100)	T1 (n = 117)	T2 (n = 100)	χ ^2^	*p*
Sex	Male	52 (52.0)	61 (52.1)	49 (49.0)	0.259	0.878
	Female	48 (48.0)	56 (47.9)	51 (51.0)		
Age group	60–69 years	38 (38.0)	48 (41.0)	42 (42.0)	3.778	0.715
	70–79 years	30 (30.0)	35 (29.9)	25 (25.0)		
	80–89 years	26 (26.0)	31 (26.5)	31 (31.0)		
	90+ years	6 (6.0)	3 (2.6)	2 (2.0)		
Education	Primary school or below	53 (53.0)	59 (50.4)	52 (52.0)	3.41	0.497
	Middle school	44 (44.0)	49 (41.9)	39 (39.0)		
	Junior college or above	3 (3.0)	9 (7.7)	9 (9.0)		
Marital status	Never married	2 (2.0)	2 (1.7)	4 (4.0)	1.923	0.771
	Married	78 (78.0)	94 (80.3)	76 (76.0)		
	Widowed or divorced	20 (20.0)	19 (16.2)	20 (20.0)		

The rates of older adults who were aware of the ongoing dementia campaign in the community were 44.0% at T0 and 47.0% at T1 (T1 vs T0, *p* = 0.683). After the OLI phase, this rate substantially increased to 73.0% (T2 vs T1, *p* < 0.001). Dementia knowledge and attitudes towards dementia scores slightly increased from T0 to T1 (12.27 ± 7.79 vs 13.51 ± 7.04, *p* = 0.256; 2.11 ± 1.57 vs 2.43 ± 1.52, *p* = 0.122) and further rose at T2 (17.63 ± 4.99, T2 vs T1, *p* < 0.001; 2.98 ± 1.18, *p* = 0.010). Screening participation rates showed only a minor increase from 18.0% at T0 to 23.9% at T1 (T1 vs T0, *p* = 0.320) but significantly increased to 46.0% at T2 (T2 vs T1, *p* < 0.001) (Table 2).

**Table 2.** **Efficacy outcomes at the end of pre-intervention (T0), traditional intervention (T1), and opinion leader intervention (T2)**.

Outcome	T0 (n = 100)	T1 (n = 117)	T2 (n = 100)	T1 vs T0		T2 vs T1	
				χ^2^/Z*	*p*	χ^2^/Z*	*p*
Awareness of the ongoing dementia campaign in the community, n (%)	44 (44.0)	55 (47.0)	73 (73.0)	0.197	0.683	15.056	<0.001
Dementia knowledge score, mean (standard deviation)	12.27 (7.79)	13.51 (7.04)	17.63 (4.99)	1.137	0.256	4.362	<0.001
Stigmatizing attitudes toward dementia score, mean (standard deviation)	2.11 (1.57)	2.43 (1.52)	2.98 (1.18)	1.546	0.122	2.569	0.010
Participation in dementia screening, n (%)	18 (18.0)	28 (23.9)	46 (46.0)	1.136	0.320	11.685	<0.001

*The knowledge and attitudes scores were skewed in their distribution, so the Mann-Whitney U test was used.

## 4. Discussion

To the best of our knowledge, only one previous dementia-related study has involved respected senior neurologists as opinion leaders in an educational intervention aimed at improving local neurologists’ adherence to dementia guidelines. The study findings support the effectiveness of OLI in improving the adoption of several practice guidelines [23]. Unlike this prior study, our study examined the feasibility and efficacy of OLI in improving dementia-related knowledge, attitudes, and participation in screening programs among a very large population of older adults residing in the same community. The successful implementation of OLI and the improved outcomes in dementia knowledge, attitudes, and participation following the OLI in this study support the feasibility of OLI in a community-based public health campaign aiming at improving the dementia-related knowledge and behaviors of the entire community’s elderly residents. These data are particularly meaningful in the context of China’s rapidly aging population and the ambitious goals outlined in the *National Action Plan for Addressing Dementia in the Elderly (2024–2030)* to enhance dementia awareness and screening rates nationally.

The significant improvements in dementia knowledge, reduction in stigmatizing attitudes, and substantial increases in screening participation rates observed at the end of the OLI phase—compared with the end of the traditional intervention phase—highlight the added value of leveraging trusted community figures to disseminate health information. Specifically, from T1 to T2, the mean dementia knowledge score rose by 4.12 points, the attitude score improved by 0.55 points, and the screening participation rate increased sharply by 22.1%. In contrast, during the T0 to T1 period following the traditional intervention, the mean dementia knowledge score rose by only 1.24 points, the attitude score improved by 0.32 points, and the screening participation rate saw only a 5.9% increase. The minimal improvements from T0 to T1 suggest a potential ceiling effect in the efficacy of traditional interventions, as progress stagnated over the 6-month period. Conversely, the statistically significant and practically meaningful increases from T1 to T2 demonstrate that the OLI approach is more effective than traditional health education in engaging the target population and disseminating cognitive health information. Further supporting this explanation is the shifting awareness rates of the dementia campaign: a slight 3.0% increase from T0 to T1, followed by a substantial 26.0% surge from T1 to T2. These patterns indicate that the broader coverage of dementia-related information among older adults—achieved through the OLI—was driven by opinion leaders, who played a critical role in reaching a larger subpopulation of elderly residents.

Several factors likely contributed to the success of the OLI. First, opinion leaders are often well respected and trusted within their communities, which can enhance the credibility of the health messages they convey [24]. Unlike passive learning through lectures or brochures, the interpersonal and interactive nature of OLI allows for tailored discussions that address individual concerns and misperceptions. This approach aligns with the principles of the Diffusion of Innovations Theory, where opinion leaders serve as early adopters and facilitators of behavioral change within social networks. Second, opinion leaders often have a wide-reaching influence in their social circles, enabling them to disseminate information effectively and encourage positive actions like seeking screening for dementia [17]. Third, the role-modeling of opinion leaders also plays an important role. By openly discussing dementia and advocating for screening, opinion leaders set an example for others to follow, thereby promoting awareness and destigmatizing the condition [25]. Finally, the training provided to opinion leaders ensured that they were equipped with accurate and practical information about dementia, enabling them to effectively communicate key messages and encourage healthy behaviors.

The present study also highlights limitations in traditional health education methods for reaching and engaging older adults, particularly in resource-intensive, large-scale public health campaigns. While lectures and the distribution of educational materials improved knowledge and awareness to some degree, these gains were modest and progressed more slowly compared with the improvements observed during the OLI phase. This disparity suggests that passive information delivery—such as one-way lectures or printed materials—may inadequately address barriers including stigma, low motivation, social isolation, and practical challenges (e.g., mobility limitations or sensory impairments). In contrast, the OLI’s interactive, personalized approach directly targets these barriers, as demonstrated by its stronger and more rapid improvements in outcomes.

However, this study has several limitations. First, to ensure questionnaire response rates, older adults were permitted to choose their preferred administration method (e.g., face-to-face interview vs online video chat). This variability in administration across the three samples may have introduced bias, as face-to-face respondents might have reported more socially desirable attitudes towards dementia compared with other modes. Second, the retrospective cohort design lacked a control group and prospective follow-up, limiting causal inferences. Observed post-OLI changes may reflect external confounders or temporal trends rather than the intervention’s effects. Therefore, the findings should be interpreted as preliminary evidence of the feasibility and potential efficacy of an OLI in dementia campaigns. Third, the study population’s representativeness is constrained by recruitment from a single urban community, cautioning against generalization to rural or socioeconomically/culturally distinct populations. Fourth, while effective identification of opinion leaders is critical to OLI success, current selection strategies may lack adaptability across diverse sociocultural contexts, potentially compromising feasibility in heterogeneous communities. Fifth, the 73.0% campaign awareness rate at T2 suggests that there are still shortcomings in the OLI approach: (a) inadequate opinion leader coverage for the elderly population, (b) a 6-month intervention period insufficient for comprehensive information dissemination, and (c) insufficient intervention intensity to reach all residents. Notably, achieving full coverage through health education or OLI likely requires extended timeframes, which may partly explain the inferior outcomes of traditional health education (conducted over a shorter duration). To address these limitations, future research should prioritize randomized controlled trials with geographically and demographically diverse populations to enhance validity. Furthermore, standardized algorithms should be developed to estimate optimal opinion leader numbers and intervention durations in community-based dementia campaigns.

To further enhance the effectiveness of OLI, several adjustments could be explored. For instance, integrating opinion leaders with other community-based strategies, such as peer support groups or technology-based interventions, could amplify their reach and impact. Moreover, leveraging digital platforms, such as local WeChat groups or community apps, to complement opinion leaders’ efforts might help to disseminate information more widely, especially among socially isolated or hard-to-reach populations [26]. Expanding the pool of opinion leaders to include individuals from diverse backgrounds, such as local business owners, religious leaders, or influential community volunteers, could also strengthen the intervention’s acceptability and penetration.

## 5. Conclusion

In conclusion, this study provides promising evidence that OLI is a potentially effective and feasible strategy to address the challenges of dementia recognition and management in China. By mobilizing trusted community figures, public health programs could overcome barriers to engagement and create a more informed and supportive environment for older adults. These findings have important implications for the implementation of China’s national dementia plan and suggest that incorporating OLI into broader public health campaigns could help achieve the stated goals of improving dementia awareness, reducing stigma, and promoting early screening. Future efforts should focus on refining the OLI model, scaling it up to other settings and evaluating its long-term sustainability and impact.

## Availability of Data and Materials

The data that support the findings of this study are available upon reasonable request from the corresponding author.
